# Assessing the population relevance of endocrine‐disrupting effects for nontarget vertebrates exposed to plant protection products

**DOI:** 10.1002/ieam.4113

**Published:** 2019-01-30

**Authors:** Mark Crane, Nina Hallmark, Laurent Lagadic, Katharina Ott, Dan Pickford, Thomas Preuss, Helen Thompson, Pernille Thorbek, Lennart Weltje, James R Wheeler

**Affiliations:** ^1^ AG‐HERA Faringdon United Kingdom; ^2^ Bayer SAS, Crop Science Division Regulatory Toxicology Sophia‐Antipolis Cedex France; ^3^ Bayer AG, Crop Science Division Environmental Safety Monheim am Rhein Germany; ^4^ BASF SE Crop Protection—Ecotoxicology Limburgerhof Germany; ^5^ Syngenta Jealott's Hill International Research Station Bracknell United Kingdom; ^6^ Present address: BASF SE, APD/EE Limburgerhof Germany; ^7^ Corteva Agriscience Agriculture Division of DowDuPont Oxfordshire United Kingdom

**Keywords:** Endocrine disruption, Plant protection product, Vertebrate population, European Union, Hazard assessment

## Abstract

The European Commission intends to protect vertebrate wildlife populations by regulating plant protection product (PPP) active substances that have endocrine‐disrupting properties with a hazard‐based approach. In this paper we consider how the Commission's hazard‐based regulation and accompanying guidance can be operationalized to ensure that a technically robust process is used to distinguish between substances with adverse population‐level effects and those for which it can be demonstrated that adverse effects observed (typically in the laboratory) do not translate into adverse effects at the population level. Our approach is to use population models within the adverse outcome pathway framework to link the nonlinear relationship between adverse effects at the individual and population levels in the following way: (1) use specific protection goals for focal wildlife populations within an ecosystem services framework; (2) model the effects of changes in population‐related inputs on focal species populations with individual‐based population models to determine thresholds between negligible and nonnegligible (i.e., adverse) population‐level effects; (3) compare these thresholds with the relevant endpoints from laboratory toxicity tests to determine whether they are likely to be exceeded at hazard‐based limits or the maximum tolerated dose/concentration from the experimental studies. If the population threshold is not exceeded, then the substance should not be classified as an endocrine disruptor with population‐relevant adversity unless there are other lines of evidence within a weight‐of‐evidence approach to challenge this. We believe this approach is scientifically robust and still addresses the political and legal requirement for a hazard‐based assessment. *Integr Environ Assess Manag* 2019;15:278–291. © 2018 The Authors. *Integrated Environmental Assessment and Management* published by Wiley Periodicals, Inc. on behalf of Society of Environmental Toxicology & Chemistry (SETAC)

## Key Points

- In this paper we consider how the European Commission's hazard‐based Plant Protection Product Regulation can be operationalized to distinguish between substances with adverse population‐level effects and those for which observed adverse effects do not translate into adverse population‐level effects.
- Our approach is to use population models within the adverse outcome pathways framework to link the nonlinear relationship between adverse effects at the individual and population levels.
- Only if the population threshold is exceeded should the active substance be classified as an endocrine disruptor with population‐relevant adversity, unless this classification is challenged by other lines of evidence (e.g., from field studies) within a weight‐of‐evidence approach.
- We believe this approach is scientifically robust and addresses the political and legal requirement for a hazard‐based assessment.

## INTRODUCTION

The potential effects of endocrine‐disrupting chemicals (EDCs) on wildlife have been a concern for many years (Campbell and Hutchinson 1998; Kidd et al. 2007; Tyler et al. 2008). Laboratory‐based studies have shown that certain chemicals can disrupt specific components of vertebrate endocrine systems in individual organisms, but the regulatory goal for environmental risk assessment is usually to protect populations of organisms (Munns et al. 2007); thus, extrapolation from laboratory to field and from individual organism to population is required.

Some studies have demonstrated a strong link between adverse endocrine effects found in the laboratory and adverse effects found in natural populations. For example, Giesy et al. (2003) and Ottinger et al. (2011) review the effects of endocrine disruptors in birds, including the effects of now‐banned organochlorine substances on gull sex ratio and clutch “superabundance,” for which there is clear laboratory and field‐based evidence. However, other studies have shown that many natural populations can compensate for endocrine‐mediated effects observed in the laboratory and thereby maintain population numbers and biomass. For example, a field study by Hamilton et al. (2014) supports the conclusion that intersex and other signs of feminization in individual fish observed in both the laboratory and the field do not necessarily result in adverse population‐level effects. They sampled roach (*Rutilus rutilus*) from different southern English rivers and assessed the genetic structure of the populations at each location. Despite widespread feminization of male roach in effluent‐contaminated rivers, there was no evidence of a correlation between effective population size and predicted exposure to estrogens. In another study with fish, Harris et al. (2011) examined the breeding ability of intersex male roach when in competition with other males for females. They found that most intersex males were able to participate in spawning, and the reproductive success of mildly intersex males was similar to nonintersex males, although moderately and severely intersex males were less successful.

Matthiessen and Weltje (2015) reviewed the effects of azole compounds and their possible involvement in masculinization of wild fish populations to determine whether there is a biologically causal link between exposure to azoles and endocrine‐disrupting effects in wild fish populations. They concluded that the available data on exposure and effects provide reassurance that reported environmental concentrations of certain azoles are insufficient to cause adverse effects in fish by interference with their endocrine system, despite results from laboratory studies that show masculinization in fish under continuous exposure at significantly higher concentrations.

Recovery from endocrine‐disrupting effects may also be reasonably rapid if the source of the endocrine‐disrupting substance is removed. For example, Kidd et al. (2007) conducted a 7‐year, whole‐lake experiment at the Experimental Lakes Area in Canada and showed that chronic exposure of fathead minnow (*Pimephales promelas*) to low concentrations (5–6 ng  ·  L^−1^) of the potent estrogen 17α‐ethynylestradiol (EE2) led to feminization and intersex in males and altered oogenesis in females, leading to near extinction of the population in the lake. However, in a follow‐up study, Blanchfield et al. (2015) quantified the physiological, population, and genetic characteristics of the fathead minnow population for 7 years after EE2 additions ceased. They found that 3 years after treatment, whole‐body vitellogenin concentrations in male fathead minnow had returned to baseline levels, there were no testicular abnormalities, and in the fourth year, adult size‐frequency distribution and abundance had returned to pretreatment levels.

Wild vertebrate populations may therefore be either resistant or resilient (for definitions, see *Adverse Effects* section) to the effects of endocrine‐disrupting substances, even when adverse effects of these substances are observed in laboratory tests. It is therefore important to establish a biologically causal link between endocrine‐disrupting effects measured in laboratory toxicity tests and adverse population‐level effects on vertebrate wildlife in the field, because this is necessary to meet the requirements of the definition of an endocrine disruptor for regulatory purposes (WHO/IPCS 2002; EC 2018). If this is not done, then useful products are likely to be banned on the basis of effects observed under laboratory conditions that are unlikely to translate to the population level in nature. It is worth noting that environmental risk assessments have been performed for decades to help prevent adverse effects on vertebrate populations, which, as shown by Matthiessen et al. (2018), may explain why the evidence for endocrine disruption due to current‐use chemicals in wildlife populations is quite limited.

In an accompanying Learned Discourses paper (Crane et al. this issue), we discuss recent European Union (EU) regulation (EC 2018) and guidance (ECHA/EFSA 2018) intended to protect humans and vertebrate wildlife populations from adverse effects due to plant protection products (PPPs) containing an EDC. In this paper we describe possible approaches that complement this guidance and would assist registrants and regulatory authorities in distinguishing between (i) substances with adverse endocrine‐mediated population effects and (ii) substances for which a potential endocrine disruption issue could be concluded from laboratory toxicity tests, but which would not translate into adverse effects on wildlife populations. This activity is in response to, and complies with, the hazard‐based approach that has been politically and legally mandated by the European Commission (EC 2018).

## ASSESSMENT OF ADVERSE POPULATION EFFECTS OF ENDOCRINE DISRUPTERS

**Figure 1:**
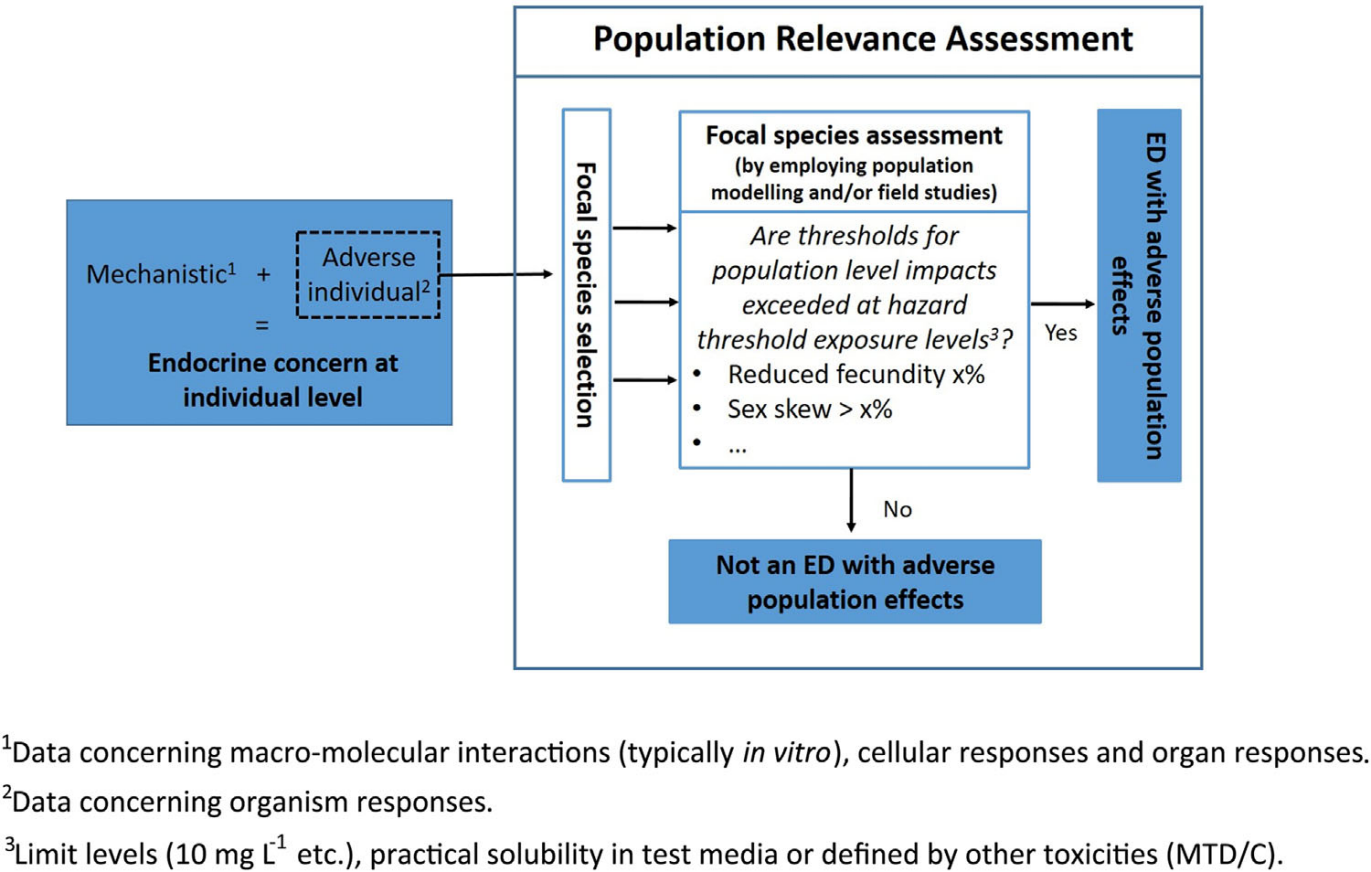
Evaluation scheme to determine the population relevance of laboratory‐determined adverse endocrine‐disrupting effects.

The European Food Safety Authority (EFSA) benefits from many expert opinions provided by their Scientific Committee (EFSA SC) and Panel on Plant Protection Products and their Residues (EFSA PPR). We draw upon these opinions here to assist in mapping out a coherent and conservative hazard‐based approach (Figure 1) for demonstrating the population relevance of endocrine disruptor effects observed in the laboratory.

The approach depends on clear identification of specific protection goals for focal wildlife populations within the EFSA's ecosystem services framework. The magnitude of relevant effects in toxicity tests performed at hazard‐based thresholds (i.e., regulatory‐defined limits or a maximum tolerated dose/concentration [MTD/C]) is compared to population‐relevant thresholds derived from population modeling. The relevance of specific endpoints is taken from an understanding of the adverse outcome pathway (AOP) concept (Ankley et al. 2010) focusing on adverse effects caused by an endocrine disruptor at the individual level that may translate to the population level. If the population threshold is not exceeded, then the substance should not be classified as an endocrine disruptor with adverse population effects unless there are other lines of evidence within a weight‐of‐evidence approach to challenge this. This approach is analogous to that for human health in which the relevance of animal model effects to humans can be challenged in the endocrine disruptor properties evaluation.

### Focal species

It is not possible, desirable, or legally permissible to test all vertebrate species in the laboratory, nor is it possible to perform risk assessments for every vertebrate species in an ecosystem. Therefore, the concept of “focal species” has been adopted from conservation biology (Lambeck 1997) to focus assessments on those species of most relevance to perceived threats. For example, in the case of PPPs these will be species that are present in specific agricultural landscapes and are thus most likely to be exposed to a PPP. Focal species may be keystone species (organisms with an effect on the ecosystem that is disproportionate to their numerical abundance), umbrella species (organisms that cover a large geographical area in their daily or seasonal movements), flagship species (charismatic organisms with public appeal), or indicator species (organisms sensitive to change and therefore useful in monitoring habitat quality). Focal species may fulfill more than one of these criteria (e.g., they may be both an indicator and a flagship species).

The concept of focal species has been adopted most fully for birds and mammals in PPP assessment, although the concept has wider application for other vertebrate taxa and has also been explored for fish (Ibrahim et al. 2013), amphibians, and reptiles (EFSA PPR 2018). Mintram et al. (2017), in a review of modeling approaches for risk assessment of endocrine disruptors in fish, recommend that “the development of future models should include species representing a range of life‐histories and…their selection should be guided by the derivation of ecological scenarios which are relevant to major land use and water body types in which chemical exposures and effects are predicted according to current risk assessments.”

EFSA (2009) distinguishes between “generic focal species” and “focal species” when assessing PPP risks to birds and mammals and defines them as follows:

A generic focal species is not a real species, however it is considered to be representative of all those species potentially at risk, that is it is based on ecological knowledge of a range of species that could be at risk. It has a high food intake rate and may consume a mixed diet rather than just one as for the indicator species. The diet is not real but is considered to be representative of the species represented …. The “generic focal species” is also considered to be a representative of the types of birds or mammals that occur across Member States.

A focal species is a real species that actually occurs in the crop when the PPP is being used. The aim of using a “focal species” is to add realism to the risk assessment insofar as the assessment is based on a real species that uses the crop. It is essential that the species actually occurs in the crop at a time when the PPP is being applied. It is also essential that this species is considered to be representative of all other species that may occur in the crop at that time. As a “focal species” needs to cover all species present in the crop, it is possible that there may be more than one “focal species” per crop.

Focal bird and mammal species are identified for specific exposure scenarios (i.e., a PPP application to a particular crop type at a specific growth stage), ideally by use of transect or field survey methods across a range of representative fields, although literature and census data might also be useful (EFSA 2009). The survey data are then used to select focal species by considering their feeding strata, food intake rate, body weight, and diet to ensure that species with the highest potential exposures are considered. EFSA (2009) notes that a focal species is not automatically the species that was most frequently seen but that it should represent the feeding guild(s) that raised concern at earlier stages in the risk assessment and represent other species.

Selection of focal species when assessing the potential population relevance of endocrine disruptors should therefore include the following considerations:

1) At least one relevant (generic) focal species should be selected for each regulatory exposure scenario (e.g., crop and application scenario). Examples of generic focal bird and mammal species are provided in EFSA (2009), and Ibrahim et al. (2013) have derived a list of focal fish species. An EFSA PPR (2018) opinion suggests that the following amphibian and reptile focal species should be considered: the great crested newt (*Triturus cristatus*), the natterjack toad (*Epidalea calamita*), the common treefrog (*Hyla arborea*), the Hermann's tortoise (*Testudo hermanni*), the sand lizard (*Lacerta agilis*), and the smooth snake (*Coronella austriaca*).
2) Each of the selected focal species should, as a priority, be potentially ecologically susceptible to adverse effects caused by exposure to endocrine disruptors (i.e., they should be indicator species). “Susceptibility” here does not mean that the species has a proven toxicological sensitivity to the substance of interest (most focal species will not have been tested in the laboratory, so we would not know). Instead it means that the ecology and demographic structure of the population make it vulnerable to the adverse endocrine‐mediated effects from the substance of interest. In this sense, the focal species acts as a surrogate for all other species in its taxonomic group because it has life history and other ecological traits at the more susceptible end of the spectrum for that taxon. Similar thinking led EFSA (2010) to suggest that a species is “vulnerable” if there is a combination of high exposure potential, particular life history characteristics, sensitivity, low dispersal ability, and low reproductive potential (i.e., a low potential for recovery).
3) The role of the selected species as a keystone, umbrella, or flagship species is of secondary importance to its ability to act as an indicator.

### Protection goals

The PPP regulation (EC 2018) uses the term “(sub)population,” which requires definition before it is possible to set protection goals. This definition has usefully been provided in the guidance, which states that “The term (sub)population is of predominant relevance with respect to humans, therefore for non‐target organisms the term population is used throughout the document” (ECHA/EFSA [2018] guidance, footnote p 4).

We therefore also use the term “population” subsequently in this paper as an operational description of any group of individuals from the same species that occurs in a particular space during a particular time, as defined by a regulatory authority.

Nontarget vertebrate wildlife populations provide a wide variety of ecosystem services including food (for human consumption), genetic resources (biodiversity), education and inspiration, aesthetic values, pest and disease regulation (e.g., birds feeding on caterpillars), seed and propagule dispersal, and recreation and ecotourism (e.g., bird‐watching, hunting, and fishing) (EFSA 2010). The EFSA SC (2016a) uses the concept of ecosystem services to derive specific protection goals (SPGs) for service‐providing units (SPUs). An SPU can be any ecological entity that provides an ecosystem service (provisioning, regulating, cultural, or supporting services) to humans. EFSA SC (2016a) states that the following need to be defined before setting an SPG: the ecological entity (e.g., individual, population, functional group, or ecosystem), the attribute of that entity (e.g., behavior, growth, abundance, biomass, or ecosystem processes), the magnitude of effects (i.e., negligible, small, medium, or large), the temporal scale of effect for the attribute (e.g., duration and frequency), and the spatial scales (e.g., in‐field and off‐field patches of landscapes). If the ecological entity to protect is the population of a particular species, then EFSA SC (2016a) suggests that in most cases the attribute to be protected will be population dynamics in terms of abundance (e.g., numbers of individuals and their fitness) or biomass. The ecosystem services approach was supported at a stakeholder workshop reported by EFSA (2010) and is now a more widely used concept in EU PPP assessment (e.g., Topping and Luttik 2017).

EFSA PPR (2014) makes the point that since the ecosystem services for SPGs for vertebrates derived in EFSA PPR (2010) are performed by populations or groups of populations, there needs to be development of appropriate population models for use in risk assessment. This view is supported by the EU guidance documents on bird and mammal risk assessment (EFSA 2009) and aquatic risk assessment (EFSA PPR 2013).

### Adverse effects

Table 1 sorts different types of effect measurements from mammalian, bird, fish, and amphibian test guidelines and places them within the framework of the AOP concept (Ankley et al. 2010) and the revised OECD conceptual framework (OECD 2018a). The AOP concept is a robust framework in which to organize such information on endocrine potential and help support regulatory decision making (Kramer et al. 2011; Becker et al. 2015; Wheeler and Weltje 2015; Edwards et al. 2016). The concept reflects the definition of an endocrine disruptor: requiring an endocrine mechanism (i.e., molecular initiating event[s]), causally linked (via key events or key event relationships) to an adverse outcome (organism or, more relevant here, population responses). The question that then needs to be addressed to meet the requirements of the PPP regulation is whether measured effects at the organism or individual‐animal level (i.e., apical effects measured in in vivo laboratory studies) translate into truly adverse effects at the population level. It is only endocrine disruptor–related effects on wildlife populations that are relevant here, and not systemic or other types of toxicity. This focus is because the PPP regulation states that “Adverse effects that are non‐specific secondary consequences of other toxic effects shall not be considered for the identification of the substance as an endocrine disruptor with respect to non‐target organisms.”

**Table 1:** Toxicological endpoint and AOP classification table for endocrine disruptor effects in vertebrates

	Adverse outcome pathway				
Taxa	Macromolecular interactions	Cellular responses	Organ responses	Organism responses	Population responses
Amphibians Cf. OECD TGs 231 and 241	Typically in vitro assessment in mammalian‐based systems but considered qualitatively relevant to all vertebrate taxa Cf. OECD TGs 455, 456, 458, and 493, US EPA OPPTS 890.1150 and 1200, and other relevant sources (e.g., EDSP21 and ToxCast assays)	• Vitellogenin	• Gross necropsy of endocrine organs • Histopathology, for example, thyroid, gonads, liver, kidney • Liver Somatic Index	• Growth • Behavior • Sex ratio • Hind‐limb length • Developmental stage • Time to metamorphosis	Potential effects on population • Size (abundance or biomass) • Stability • Recruitment
Birds Cf. OECD TG 206			• Gross necropsy of endocrine organs	• Reproduction (fecundity, fertility) • Growth • Behavior • Hatching success • Eggshell thickness • Number of 14‐day survivors	
Fishes Cf. OECD TGs 229, 230, 234, and 240 and modifications of US EPA OPPTS 850.1500		• Vitellogenin • Hormone levels	• Histopathology, for example, thyroid, gonads, liver, kidney • Secondary sexual characteristics • Gonadal Somatic Index	• Reproduction (fecundity, fertility) • Time to maturity • Growth • Behavior • Sex ratio • Embryo time to hatch • Hatching success	
Mammals		• Hormone levels • Steroidogenesis (gene or enzyme changes) • Sperm morphology • Sperm motility • Sperm numbers • Vaginal smears	• Gross necropsy of endocrine organs • Histopathology, for example thyroid, gonads, liver, kidney • Secondary sexual characteristics • Sexual maturity landmarks, such as –Age at first estrus –Estrus cyclicity –Age at balanopreputial separation –Age at vaginal opening –Nipple development –Anogenital distance • Keratinization and cornification of vagina • Proliferation of endometrial epithelium (colloid area and follicular cell height)	• Reproduction (fecundity, fertility) • Time to maturity • Growth • Behavior • Sex ratio • Dystocia • Fetal development • Gestation length • Litter size • Litter viability • Litter or pup weight • Number of implantations, corpora lutea	
Data considerations (causal links must be established between responses at different biological levels)	OECD CF level 1 + 2Mechanistic only, not indicative of adversity	• OECD CF level 3, 4 + 5 Mechanistic only, not indicative of adversity	• OECD CF level 3, 4 + 5 Predominately mechanistic, may give an indication for potential adversity	• OECD CF level 4 + 5Adverse at the individual level. If observed and confirmed in OECD CF level 5 studies, in the absence of additional data, assumed relevant at the population level, that is, assumed by default to be relevant (cf. endocrine disruptor criteria).	Population responses unpredictable unless • Simulated by population modeling, or • Derived empirically from semifield or field studies

The concept and definition of an “adverse effect” has received considerable attention in toxicology (Kerlin et al. 2016). For example, Lewis et al. (2002) state that an effect is unlikely to be adverse if:

1) There is no alteration in the general function of the test organism or affected organ or tissue;
2) It is an adaptive response;
3) It is transient;
4) The severity is limited and below thresholds of concern;
5) The effect is isolated or independent;
6) The effect is not a precursor to adverse effects;
7) It is secondary to other adverse effect(s); or
8) It is a consequence of the experimental model.

In addition, an effect is unlikely to be adverse if the response lies within historical control ranges (e.g., Valverde‐Garcia et al. 2018). For an effect to be considered adverse in an environmental context it is normally assumed to be a measure that is evident at the whole‐animal level, whereby the suborganism responses are integrated into an apical effect measure (e.g., growth, development, or reproduction). This integration is not intended to diminish the value of suborganism responses in an understanding of the overall toxicological response, especially in terms of establishing an endocrine mechanism and, potentially, as indicators for adversity. However, it is the organism responses (see Table 1, ‘“organism response” column) that provide the individual adverse response variables that will also serve as input variables for population modeling. This thinking aligns with the currently accepted testing paradigm in which the more definitive eco/toxicological assays sitting at level 5 of the OECD Conceptual Framework (OECD 2018a) are typically used to confirm whether endocrine activity identified at lower levels translates into endocrine‐mediated adverse effects.

EFSA SC (2013) identifies 3 aspects of a nontarget population that should be addressed when assessing the possible adverse effects of an endocrine disruptor:

1) Population recruitment;
2) Population size; and
3) Population stability.

They also state that adverse consequences on reproduction, growth and development, disease incidence, and survival in one or more species should be addressed “as these are the effects most likely to impact on population recruitment and stability”, although we note that survival is not an endpoint of relevance to endocrine disruption and, to the best of our knowledge, disease incidence is rarely considered explicitly in chemical risk assessment.

The EFSA (2009) guidance document on bird and mammal risk assessment mentions that although the magnitude of an effect in an exposed group could be statistically significantly different to that in the controls, it might not be biologically relevant, and states:

In order to determine the biological relevance of an effect it should be considered whether the effect could lead to a functional deficit later on in the study, e.g. if a reduction in the weight of pups at birth leads to a decrease in level of survival. If not, then the effect may not be biologically relevant, however if there is a carry‐over of effects into the number of survivors, it can be considered biologically relevant.

For example, in 2‐generation mammalian reproduction studies, a body‐weight reduction in pups would not be considered relevant if normal development was observed in the F1 generation, especially if F1 fertility and reproduction were comparable to the control (EFSA 2009). Similarly, a reduction in sperm production in rodents with multiple matings may not adversely affect reproduction.

The EFSA SC opinion on the hazard assessment of endocrine disruptors (EFSA SC 2013) argues that the concept of “biological relevance” is based on the assumption that a “normal” biological state can be defined. In turn, “normality” is linked to the adversity of an effect observed during toxicity testing or in epidemiological studies. The SC states that the point at which endocrine modulation becomes an adverse effect cannot be based on an absolute response value but only on a relative response (compared to the control or background response).

EFSA SC (2017a) suggests that models can be used for setting a critical effect level (i.e., a benchmark response [BMR]). They envisage that models

of focal species could be used to determine endpoints corresponding to cut‐off values set by specific protection goals (SPG). These models can be used for calculating critical effect levels for certain types of effect, for instance for egg cracking, number of surviving chicks or the size of litters, above which the population of the focal species will be negatively affected to such an extent that the population will decline over time.

**Figure 2:**
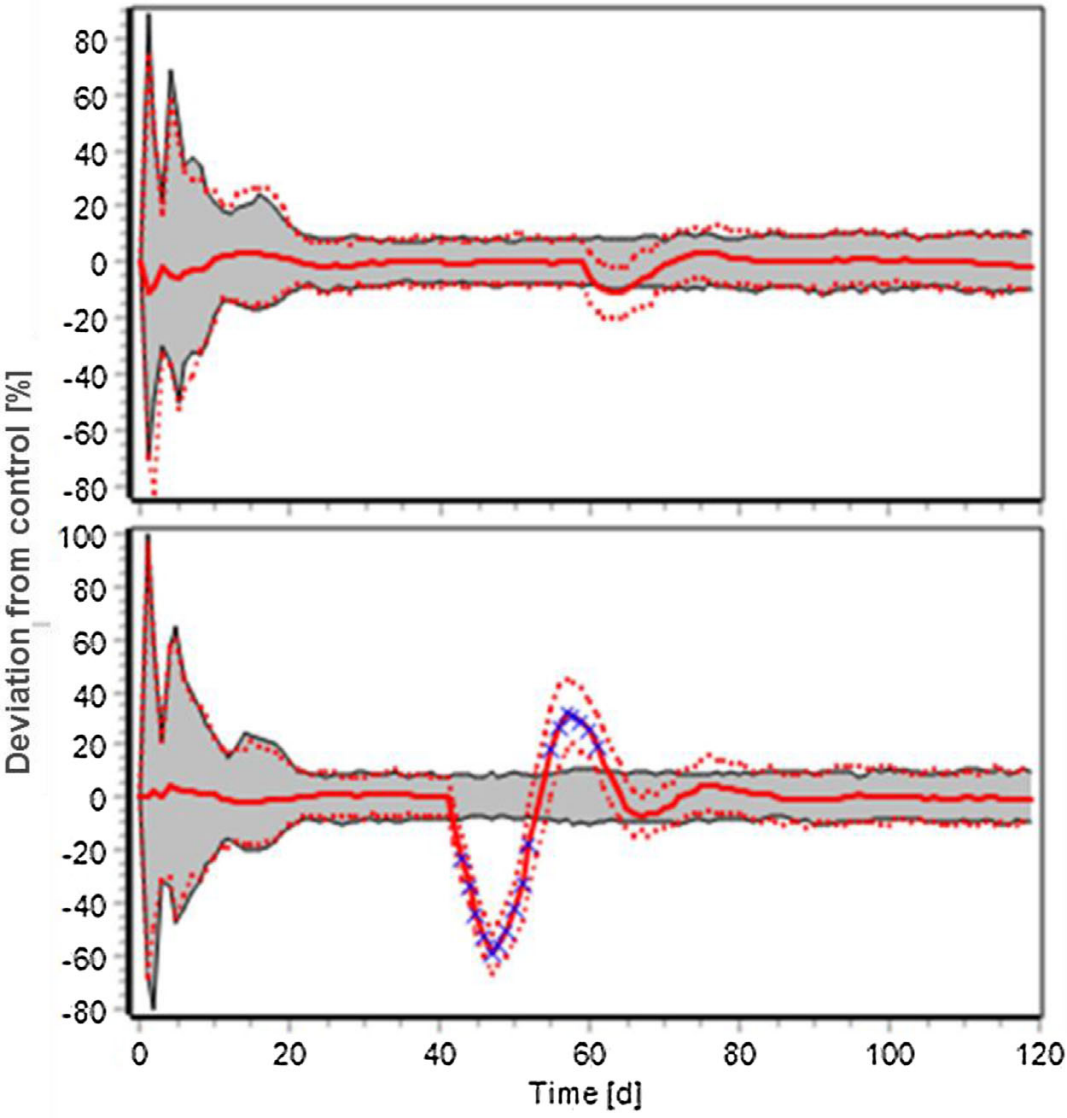
Illustration of population resistance (upper panel) and population resilience (lower panel). The gray area represents the normal operating range of the population parameter.

EFSA SC (2016b) also suggests that the concept of “recovery” may be useful when considering biological effects. Recovery is the return of the perturbed (ecological) endpoint (e.g., species composition, population density) to the “window” of natural variability as observed in the undisturbed state of the ecosystem of concern (e.g., before the stressor event took place) or to the level that is not significantly different from that in control or reference systems. They note that a system that has been subject to an adaptive response or to recovery might not necessarily return to the same state that it exhibited before the disturbance. Individual organism recovery from transient effects is a recognized concept under the PPP regulation. However, “recovery” is unlikely to be an acceptable regulatory approach to endocrine disruptor effects on vertebrate populations (EFSA PPR 2013; EFSA SC 2016b), so we do not explore it further in this paper. Instead we use the related concepts of resistance and resilience, which are of considerable value because they are key components of population stability and allow discrimination between relevant and negligible effects at the population level. EFSA SC (2016b) defines population resistance as the magnitude of environmental perturbation a population can tolerate without being pushed out of its normal operating range (i.e., a population's capacity to remain unaffected). Population resilience is related to the return time to equilibrium following a perturbation (EFSA SC 2016b and references therein). Figure 2 illustrates both these concepts. EFSA SC (2016b) specifies that population resilience depends on the ecological context and is related to the degree to which induced fluctuations in the population density are buffered by density‐dependent feedback mechanisms and competition with other species (e.g., Knillmann et al. 2012).

This definition is related to the EFSA SC (2017b) view that between individual effect reversibility and population recovery, there is also the concept of what they describe as “population relevance.” For example, small effects on fecundity in density‐regulated systems (e.g., a slightly reduced number of eggs for fish that produce many more eggs than can possibly develop into juvenile fish) will not translate adversely to the population level. EFSA SC (2017b) concludes that as, under these circumstances, there is no effect at the population level, there is no need for recovery (e.g., Hamilton et al. 2014). In other words, the population has shown resistance.

### Population modeling

We propose, in agreement with EFSA SC (2017a), that population models should be used to determine what percentage effect observed in a laboratory toxicity test exceeds a threshold percentage that translates into a nonnegligible reduction in population recruitment, size, or stability across a representative “worst‐case” landscape. We define a negligible effect as one that is buffered by population resistance so that population recruitment, size, and stability remain within their normal operating ranges (Figure 2). These normal operating ranges may be determined from historical data, model simulations, or a combination of the 2.

There are established methods to demonstrate the relevance of effects with population models to extrapolate from the laboratory to the field (e.g., Wang 2013; Liu et al 2013; Schmitt et al. 2016; Topping and Elmeros 2016; Topping and Weyman 2018). Mintram et al. (2017) reviewed modeling approaches potentially applicable to the environmental risk assessment of endocrine‐active substances in fish and concluded that individual‐based models (IBMs) are a particularly useful model type because they can account for species‐specific traits and behaviors (e.g., breeding behaviors) and simulate interorganism interactions and organism–environment interactions (including responses to chemical exposure). IBMs therefore capture both the direct and indirect population‐level effects of chemical exposures. Mintram et al. (2017) identify the main challenge as striking a balance between site‐specific versus generic applicability, because of the often complex and environmentally plastic life histories of fish.

Hazlerigg et al. (2014) developed an IBM for zebrafish based on empirical data (e.g., growth, reproduction, and mortality) derived from a combination of laboratory and field experiments, literature values, and ecological theory. The IBM was validated against size distributions for wild populations of zebrafish sampled in Bangladesh. Sensitivity analysis showed that population abundance was most sensitive to changes in density‐dependent survival and the availability of refugia for juveniles. The model was then used to determine the population‐level relevance of changes in sex ratio caused by androgenic (dihydrotestosterone [DHT]) and estrogenic (4‐*tert*‐octylphenol [4‐tOP]) substances. Both substances were investigated under acute (10‐day) and chronic (1‐year) exposure regimes. Acute exposures to the test substances had little effect on population‐level endpoints at any of the concentrations tested. Chronic exposures decreased population abundance at higher concentrations for both substances and most strongly with DHT, owing to the sex ratio shift to fewer females, which lowered population reproductive output. However, these concentrations considerably exceeded environmentally realistic levels. The model predicted that a substance‐induced change in sex ratio from 50:50 to approximately 40:60 in favor of males (DHT) or females (4‐tOP) would have limited effects on population abundance after chronic exposure but that a large reduction in abundance would occur if the ratio changed to approximately 20:80 in favor of either sex. This study suggests that an SPG for fish population abundance would be met at a benchmark concentration that did not skew sex ratios to more than approximately 40:60.

Hamilton et al. (2016) consider further examples for fish in a critical review of population‐level consequences for wild fish exposed to sublethal concentrations of chemicals. They conclude that IBMs such as those developed by Hazlerigg et al. (2014) include many more of the observed effects of chemical exposure on individuals than is the case for matrix population models and that they are useful for exploring which effects on individuals are likely to have the greatest effect on populations.

Uncertainty analysis reported with the results of population modeling helps identify and prioritize the uncertainties associated with the assessment inputs and methodology used (EFSA SC 2018). However, the use of additional ecological knowledge via population modeling of focal species can only reduce assessment uncertainty from current levels. The ECHA/EFSA (2018) guidance is unclear as to how an applicant should report the results of population models or lines of evidence other than the results from laboratory toxicity test endpoints. This ambiguity is because the reporting template associated with the guidance does not include any endpoint types other than those from laboratory tests. However, EFSA PPR (2014) provides advice on good modeling practice specific to ecological models, including a template for model summary documents and a checklist for risk assessors. These suggestions are clearly applicable when establishing the population relevance of any endocrine disruptor effects via modeling and would be suitable for submission with the endocrine disruptor reporting template. Raimondo et al. (2018) also provide useful advice on population models from the perspective of regulatory authorities.

### Field studies

EFSA/ECHA (2018) guidance states that field studies cannot be used to override or dismiss evidence of adversity found in laboratory studies. However, this contrasts with previous EFSA (2009) guidance, which provides the following main points on the use of field studies:

• Field studies of mortality and reproductive effects are neither simple nor inexpensive, but they have some important advantages over other study types:
  – They focus on the direct measurement of the effects of concern under realistic field conditions, so can take account of all routes of exposure and all relevant sources of variation.
  – They avoid uncertainties associated with extrapolation from models or laboratory studies to the field.
  – They reduce uncertainties associated with extrapolating sensitivity (toxicity) from studied species to those exposed in the field.
• Design of a study of appropriate power requires knowledge of the levels of effects that are considered acceptable and the degree of certainty that is required to prevent the acceptable limit being exceeded.
• “Extensive” approaches across a large number of sites to cover a broad spectrum of use conditions are preferable to “intensive” studies across a small number of sites, because they account for natural variability in exposure and effects.
• The choice of methods should be driven by the study objectives and might include:
  – Capture‐mark‐release‐recapture studies to monitor population changes, which include changes in age structure, especially in small mammals.
  – Monitoring of sublethal effects with biomarkers (e.g., enzyme inhibition). Repeated sampling from the same individuals may be desirable to control for high natural variability in biomarker levels, although this approach must be balanced against the risk that repeated capture will alter the behavior of the animals and hence will bias the results.
  – Visual observations to monitor populations and activity of birds and large mammals. Interpretation of results is difficult if the animals are not individually marked.
  – Monitoring of reproductive performance of birds.

EFSA (2009) guidance suggests that a well‐performed field study should carry considerable weight when the population relevance of endocrine disruptor effects on individual vertebrates is assessed. Such evidence should therefore be a powerful line of evidence, which may be used either to confirm or refute laboratory evidence of endocrine disruptor effects. This suggestion would be more consistent with the PPP regulation mandate that “Adequate, reliable and representative field or monitoring data and/or results from population models shall as well be considered where available.”

In support of this, EFSA SC (2013) states that the use of field data is valuable in providing greater confidence about whether an adverse endocrine‐mediated effect is likely to have consequences at the population level. Indeed, they go further than this:

In the absence of such data (i.e., field study data), regulators must be confident of being able to extrapolate from laboratory data on endpoints such as growth and reproduction to potential effects on populations, ideally but not necessarily through the use of population modeling.

And,

Field monitoring is probably the most powerful tool in establishing impacts at the population‐level (e.g., population declines).

We agree with the EFSA SC opinion that, when available, the use of field studies, including field monitoring, should form an important line of evidence for assessing the population relevance of an endocrine disruptor, especially when used in combination with population modeling. Field studies can also provide invaluable inputs to modeling either for parameterization or validation, and models can add to field studies by extrapolating to untested conditions. If adequate, reliable, and representative population models and field studies show that there is no evidence for an adverse population‐level effect from an endocrine disruptor then this should override laboratory data, which is a position consistent with the PPP regulation.

### Linking laboratory effects to wildlife populations

Results from laboratory tests can currently be linked to potential adverse effects at the population level either quantitatively by use of a population model, or qualitatively by use of expert judgment to bring together different lines of evidence (e.g., laboratory and field data) in an overall weight‐of‐evidence approach, as described by EFSA SC (2017b).

The population modeling for focal species described earlier, potentially complemented by data from field studies, would provide a set of simulations that show what percentage effect on an input parameter leads to a nonnegligible effect on population recruitment, size, or stability. These values from population models then provide benchmarks against which an assessor can compare the results for specific laboratory test endpoints such as eggshell cracking in a bird toxicity test. This is likely to be a doubly conservative approach if the focal species chosen for modeling is ecologically sensitive and the laboratory species chosen for testing over a prolonged chronic duration is toxicologically sensitive.

A wide range of potentially endocrine‐related endpoints are routinely measured in laboratory tests to support the registration of PPPs (Day et al. 2018). Marty et al. (2017) summarize the main endpoints measured in laboratory toxicity tests designed to assess the effects of endocrine‐active substances. They point out that most of these are “apical” endpoints such as growth, development, sex ratio, and reproduction, which can potentially be linked to adverse population effects. Increasingly, as in mammalian toxicology, historical control data are also available to allow a more thorough understanding of any statistically significant differences in these apical endpoints (e.g., Valverde‐Garcia et al. 2018).

**Figure 3:**
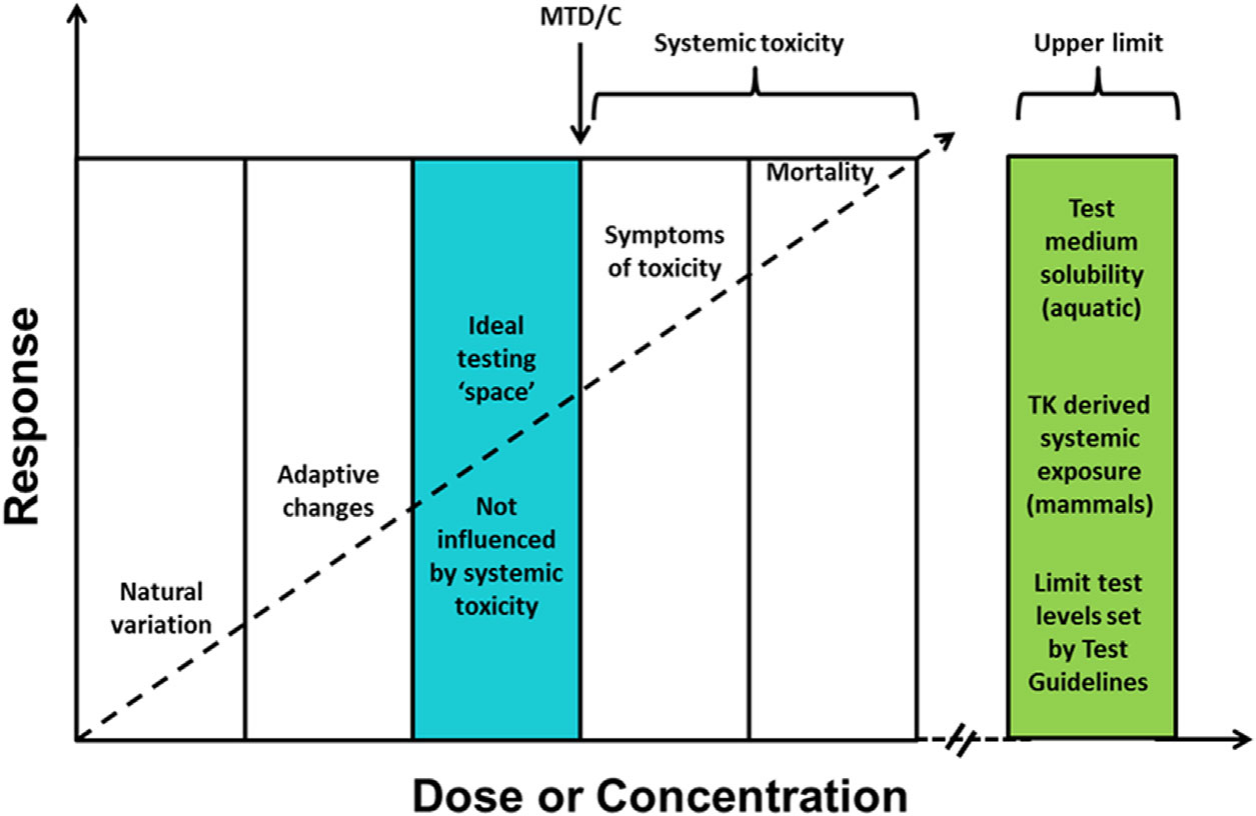
The MTD/C concept and upper test level setting that can be applied to endocrine test guideline studies (adapted after Wheeler et al. 2013). Limit test concentrations are 10 mg ·  L^−1^ for fish and aquatic amphibians, 1000 ppm diet for birds (according to OECD TG 206), and 1000 mg ·  kg^−1^ body weight  ·  day^−1^ for mammals (according to OECD TG 416).

ECHA/EFSA (2018) guidance requires that an assessment for endocrine‐disrupting properties is performed first for human health with use of the mammalian toxicology data set. The guidance suggests that this requirement is primarily based on the results from tests on rats, such as the extended one‐generation reproductive toxicity study (EOGRT; OECD 2018b, with cohort 1a/1b, including the mating of cohort 1b to produce the F2 generation) (although this study is not currently a core data requirement for PPP registration in the EU) or the two‐generation reproduction toxicity test (OECD 2001). If the criteria for establishing a substance as an endocrine disruptor are not met for human health, then the assessment should move on to consider fish and amphibians, which may require further testing (the mammalian data package could be used to assess population relevance for wild mammals, but nothing is stated about this in the guidance). The guidance suggests that this testing might primarily include the medaka extended one‐generation test (MEOGRT; OECD 2015a) and the larval amphibian growth and development assay (LAGDA; OECD 2015b), although again these are not currently data requirements for PPP registration in the EU. Any existing data on birds and reptiles should also be considered, although the guidance does not identify specific tests for these taxa, and regulatory tests do not currently exist for reptiles. Whichever test is used, the ECHA/EFSA (2018) guidance recommends that vertebrates should be tested at concentrations or doses that do not exceed an MTD/C so that systemic toxicities that may potentially confound endocrine responses are avoided. Although well established in mammalian toxicology, the MTD/C concept is relatively new to ecotoxicology (Hutchinson et al. 2009; Wheeler et al. 2013). Test levels may also be limited by the practical limit of solubility in aquatic test media because tests should not be conducted above this level (OECD 2000). If the MTD/C and water solubility are not limiting, tests should be performed at regulatory limit concentrations (e.g., chronic aquatic toxicity at 10 mg/L [OECD 2012] and bird toxicity at 1000 ppm diet [OECD 1984]). Limit test levels for certain taxa are intrinsically linked to hazard assessment because they are levels specified as classification categories for hazardous to the environment (e.g., UN 2011). This approach is illustrated in Figure 3.

## CASE STUDY

A study reported recently by Topping and Luttik (2017) illustrates the value of the approach described above. They simulated the effects of PPP exposure on populations of skylark (*Alauda arvensis*) across Danish agricultural landscapes in a study explicitly designed to show how the results of population modeling might be used to establish SPGs for this focal species. An IBM for skylarks was used to examine several scenarios that represented the range of agricultural landscapes across Denmark. In this section we describe the way in which the information in Topping and Luttik (2017) can be used to select focal species and protection goals, define adverse effects, perform population modeling, and link laboratory effects to wildlife populations.

### Focal species

The skylark is a small ground‐nesting passerine that typically feeds on insects and breeds in fields during periods of PPP application. It is also one of the farmland bird species in western Europe that has experienced a large population decline, and it is the subject of an EU management plan (Topping and Luttik 2017). The skylark therefore fulfills EFSA (2009) criteria for a focal species because it occurs in the crop when PPPs are being used and it represents other bird species that may also occur in the crop at that time.

### Protection goals

EFSA SC (2016a) guidance states that the following need to be defined before setting a protection goal: the ecological entity (e.g., individual, population, functional group, or ecosystem), the attribute of that entity (e.g., behavior, growth, abundance, biomass, or ecosystem processes), the magnitude of effects (i.e., negligible, small, medium, or large), the temporal scale of effect for the attribute (e.g., duration and frequency), and the spatial scales (e.g., in‐field and off‐field patches of landscapes). If the ecological entity to protect is the population of a particular species then EFSA SC (2016a) suggests that in most cases the attribute to be protected will be population dynamics in terms of abundance (e.g., numbers of individuals and their fitness) or biomass. Topping and Luttik (2017) examined the effects of PPP application on total skylark population size and relative abundance occupancy, a measure that compares changes in spatial coverage (occupancy) and density (abundance) with or without PPP application.

Although the specific protection goal for effects on population abundance could be set at any level, it is highly likely that regulators in the EU would insist that there should be negligible long‐term adverse effects on abundance across the most ecologically sensitive landscapes.

### Adverse effects

EFSA SC (2013) identifies population recruitment, size, and stability as the 3 aspects of a nontarget population that should be addressed when assessing the possible adverse effects of an endocrine disruptor. The skylark model described by Topping and Luttik (2017) simulates population size over a 30‐year period, so population stability and recruitment are also addressed within the modeling process.

### Population modeling

Topping and Luttik (2017) used the ALMaSS skylark model, which is an IBM within the open source Animal Landscape and Man Simulation System (https://gitlab.com/ChrisTopping/ALMaSS_all). Individual birds were categorized into 5 life stages: clutch, nestlings, prefledglings, males, and females. Available insect food biomass was determined by vegetation structure in the landscape and by its availability to birds foraging within a home range. Insect biomass was updated daily in the model and was affected by vegetation growth and human management, such as PPP application. During the breeding period the model considered the energetic balance of the adults, food requirements for maintenance, requirements of the young, and weather constraints both as a limit to foraging success and as increased energetic costs for cold weather. Initiation of breeding depended upon a bird finding a suitable territory and vegetation suitable for nesting. Breeding success depended on the habitat being able to fulfil the energetic requirements of the birds during the breeding period and the survival of eggs and nestlings, which was determined by food resource quantity and availability (a function of management, weather, and skylark behavior).

Ten model landscapes were selected to be used in the simulation runs, representing the range of agricultural practices in Denmark, from intensive to extensive. A wide range of different PPP application timing scenarios and levels of effect on different life stages were simulated.

These simulations showed that in the most sensitive of the 10 modeled landscapes a reduction in skylark eggshell thickness, which was associated with a 10% or less probability of clutch loss, led to negligible effects on population recruitment, size, and stability after a period of 20‐30 years.

### Field studies

The model used by Topping and Luttik (2017) has been extensively tested and reproduces a range of real‐world skylark population and individual behaviors. These attributes include the mean and variation around time to hatch and nest leaving, densities of skylarks per farm, and within‐season phenology under different field conditions.

### Linking laboratory effects to wildlife populations

Bird eggs begin to crack in the laboratory when shell thickness is reduced by 18% (EFSA 2009). Topping and Luttik (2017) show that a cracking rate of 10% or less did not lead to adverse population effects for skylarks in even the most sensitive simulated scenario that they investigated. A shell thinning rate above 18% but below a value corresponding to 10% cracking could therefore be set as a benchmark response.

The results for eggshell thinning and cracking from laboratory‐based avian reproduction tests can be directly linked to effects on skylark population recruitment, size, and stability, with this benchmark response indicating an inflection point below which no adverse population effects would be expected. If avian testing is performed at the hazard‐based dietary limit of 1000 ppm and the benchmark response is not exceeded, then the tested substance is unlikely to cause adverse population effects in birds.

## CONCLUSIONS

In this paper we have suggested how to implement the new endocrine disruptor criteria in the PPP regulation in a way that builds upon ECHA/EFSA (2018) guidance and is consistent with the PPP regulation legal text that mandates a hazard‐based approach. Our recommended approach is

1) Use SPGs for focal wildlife populations within EFSA's ecosystem services framework. The SPGs for vertebrate wildlife can be summarized as (i) ecological entity: populations of vertebrate focal species; (ii) attribute: population recruitment, size, and stability; (iii) effect magnitude: negligible; (iv) temporal scale: species life cycle, life span, or breeding period and an exposure frequency or duration agreed with regulatory authorities; (v) spatial scale: likely to be a watershed or landscape, but this element needs to be agreed upon with regulators; and (vi) degree of certainty: high (e.g., 95%).
2) Model the effects of changes in population‐related inputs on focal species populations with IBMs to determine thresholds between negligible population effects and nonnegligible (i.e., adverse) population effects.
3) Compare these thresholds with the relevant endpoints from laboratory toxicity tests to determine whether they are likely to be exceeded at the hazard‐based limits or the MTD/C from the experimental studies.
4) If the population threshold is not exceeded, then the substance should not be classified as an endocrine disruptor with adverse population effects unless there are other lines of evidence within a weight‐of‐evidence approach to challenge this.

If the proposals in this paper are accepted by stakeholders, then it is likely that future technical work will need to concentrate on 4 main areas: (i) Selection of a small number of ecologically sensitive focal species for each taxonomic group. This task has already been performed by EFSA for birds and mammals (EFSA 2009), and Ibrahim et al. (2013) and EFSA PPR (2018) have begun the process for fish, amphibians, and reptiles (although there are currently no laboratory test designs for the latter group). (ii) Development of suitable IBMs for these focal species. Models are already available for some bird and mammal focal species and for some fish species. However, additional effort will be required to develop models for additional focal species identified by regulatory authorities and to adapt available models for the specific questions in the endocrine disruptor hazard assessment. It may also be possible to use model outputs to develop look‐up tables for adverse population effects, which could standardize and simplify the process, but this would need to be discussed with regulatory authorities. (iii) Definition of MTD/C for all vertebrate test methods so that agreed hazard thresholds are available to minimize animal testing and reduce the likelihood of observing systemic rather than endocrine disruptor‐related toxicity in laboratory tests. (iv) Perform detailed case studies to pilot the approach.

The identification of endocrine disruptors in PPPs under the regulation is primarily a hazard‐based and not a risk‐based methodology, which is an approach to regulation that has been strongly criticized in the past (Crane et al. https://setac.onlinelibrary.wiley.com/doi/10.1002/ieam.4116). The approach to identify adverse endocrine‐disrupting population‐level effects that we recommend in this paper is hazard based to comply with the PPP regulation, but uncertainty would be significantly reduced and assessment relevance improved if exposure was also explicitly considered during the process. For example, models are available at the organism level to predict whether an endocrine‐disrupting effect would be transient under realistic patterns of exposure (e.g., toxicokinetic and toxicodynamic models and dynamic energy budget models). Use of such models might negate the need to move along an AOP to the population level by showing that an adverse endocrine‐disrupting effect had not occurred at the organism level. However, use of exposure duration and profiles information in these models implies a risk‐based approach, which is not currently allowed under the PPP regulation. We believe that further discussion of this issue between all stakeholders would help to reduce regulatory uncertainty and provide a more robust and science‐based solution to the problem of detecting and preventing adverse endocrine disruptor effects in the environment. Regardless, all substances shown to be non–endocrine disrupting and potentially endocrine disrupting, but without population‐relevant adverse effects, will still undergo risk assessment, thus ensuring a high level of protection for the environment.

## Disclaimer

Mark Crane received funding from the European Crop Protection Association (ECPA) to research and write this paper. All other co‐authors work for agrochemical companies as indicated by their affiliations.

## Data Availability

Please contact the corresponding author, Mark Crane (mark.crane@ag-hera.com), for any data used in this study.
